# Editorial: Cardiovascular diseases related to diabetes and obesity – volume II

**DOI:** 10.3389/fendo.2022.1044326

**Published:** 2022-10-05

**Authors:** Jamie L. Young, Ying Xin, Mohanraj Rajesh, Lu Cai

**Affiliations:** ^1^ Department of Pharmacology and Toxicology, University of Louisville School of Medicine, Louisville, KY, United States; ^2^ Key Laboratory of Pathobiology, Ministry of Education, Jilin University, Changchun, China; ^3^ Department of Pharmacology and Therapeutics, College of Medicine and Health Sciences, United Arab Emirates University, Al Ain, United Arab Emirates; ^4^ Pediatric Research Institute, Department of Pediatrics, the University of Louisville School of Medicine, Louisville, KY, United States

**Keywords:** metabolic syndrome, obesity, cardiovascular disease, multifactorial, biomarkers

Metabolic diseases are multifactorial, multifaceted, and even intertwined making understanding the etiopathogenesis of such diseases more complex than originally thought. This concept of intimately intertwined health conditions is exemplified in the relationship between obesity, diabetes, and cardiovascular diseases (CVDs), of which the prevalence of all three diseases are growing in parallel with one another. Nearly tripling in prevalence since 1975, obesity is considered a leading global health concern and continues be the number one risk factor for diabetes and CVDs (1). Despite our advances at the bench and in the clinic, approximately 17.9 million people die each year from CVDs, accounting for 32% of all deaths globally (2). Therefore, it is imperative to rigorously investigate the underlying mechanisms involved in the pathogenesis of obesity- and diabetes-related cardiovascular events to develop more effective, targeted therapies aimed at prevention and attenuation of disease progression. Reflective of the significance of this topic, a second volume of this special issue has been created, further compiling recent clinical, pre-clinical and basic research studies as well as review articles aimed at filling the knowledge gaps and expanding our understanding of the complicated and intertwined relationship between obesity, diabetes, and CVDs.

The multifactorial, intertwined nature of metabolic diseases has become increasingly apparent. In fact, the term “metabolic syndrome” is used to describe a group of conditions that work together to increase the risk of diabetes and CVDs. It is pertinent to note obesity is the main risk factor for metabolic syndrome. A major contributor to CVDs is premature myocardial infarction (PMI), referring to myocardial infarction that occurs in men less than 55 years old and women less than 65 years old. In an observational study by Gao et al. (2022) 772 patients less than 45 years old diagnosed with acute myocardial infarction were divided into groups based on whether they had metabolic syndrome or not and followed up for approximately 42 months for major adverse cardiovascular events (MACE). Metabolic syndrome was an independent risk factor for MACE with hyperglycemia being an independent predictor for both coronary artery lesions and MACE. These results highlight the complex association between metabolic diseases as well as the need for early intervention in both metabolic syndrome and PMI.

Further highlighting the multifactorial traits of cardiovascular diseases, Wen et al. (2022) demonstrated the increased risk of cardiovascular complications, specifically coronary heart disease (CHD), associated with obstructive sleep apnea hypopnea syndrome (OSAHS) are related to inflammatory factors, glycol-lipid metabolism, obesity status, and HOMA-IR. In patients with OSAHS and CHD there was a significant increase in triglycerides, serum inflammatory factors C-reactive protein (CRP), tumor necrosis factor-α (TNF-α), interleukin-6 (IL-6), and interferon-γ (IFN-γ) when compared to patients with OSAHS alone. Results from this study emphasize the inseparable relationship of OSAHS with inflammatory factors, obesity status and heart disease, demonstrating the importance of considering the influence of these factors in prevention and treatment of OSAHS.

The multifactorial pathogenesis of diabetic cardiomyopathy is eloquently reviewed in the article by Chen et al. (2022) highlighting the involvement of key cellular networks involved in inflammation and oxidative stress that result in abnormal lipid and glucose metabolism (a key characteristic of obesity) and ultimately diabetic cardiomyopathy. The potential pathways by which glycol- and lipid-metabolism disorders damage the diabetic heart are substantial. Detrimental alterations in cellular events that contribute to dysregulated lipid and glucose metabolism, such as the up regulation of CD36, the AGEs/RAGE system, hexamine biosynthesis and NLRP inflammasome activation, have all attracted considerable research attention making them potential targets for therapeutic interventions for not only diabetic cardiomyopathy, but also chronic inflammatory diseases and cancer. With such advances, there is a need for researchers to reevaluate the exact effects of the established, first-line drugs (i.e., metformin and thiazolidinediones) on diabetic cardiomyopathy.

An alternative approach to developing new drugs is the repurposing of established, already approved therapies, a strategy that is gaining traction because it builds on already available information including structure, formulations and potential toxicity while decreasing cost and improving the rates of clinical success. This approach was applied in an integrated bioinformatics study by Guo et al. (2022) who identified 5 potential immune biomarkers in diabetic cardiomyopathy patients using existing microarray data from the Gene Expression Omnibus (GEO) database. Expression levels of the 5 immune biomarkers were confirmed in an animal model of diabetic cardiomyopathy, with endothelin-1 (EDN1) showing significant expression differences in both the dataset and animal model. The authors then screened for potential, known therapeutic drugs that could regulate EDN1 upstream transcription factors and identified 9 candidate compounds for potential drug repurposing followed by molecular docking simulations. This data is compelling, suggesting EDN1 is a potential biomarker of diabetic cardiomyopathy while highlighting the potential of drug repurposing, which needs urgent validation using robust *in vivo* models. In support of this notion, Wang et al. (2022) targeted endothelial dysfunction, an early pathological event in diabetic angiopathy, with curcumin, a natural polyphenol shown to attenuate diabetes-associated endothelial dysfunction, and baicalein, a phytochemical shown to inhibit vascular inflammation. Both *in vivo* and *in vitro* results revealed curcumin and baicalein synergistically restored endothelial cell survival and blood vessel structure repair as well as decreases blood sugar and lipid levels in rats. Although this study did not focus on diabetic cardiomyopathy, this novel combination of therapies on the endothelial system creates a platform for investigating this combination therapy for all diabetic CVDs.

The discovery of novel biomarkers of CVDs is key to the advancement of preventative measures and disease treatment. Yang et al. (2022) investigated the role of postprandial hyperglycemia in the pathogenesis of coronary artery disease in 2970 Chinese patients who underwent coronary angiography. Patients were categorized by whether they had diabetes or not and 1,5-Anhydroglucitol (1,5-AG) was measured in their serum. In contrast to hemoglobin A1c (HbA1c) (reflective of average blood sugar levels over the past 2-3 months), 1,5-AG reflects short-term, circulating glucose fluctuations, thus predictive of postprandial hyperglycemia. The prevalence and severity of coronary artery disease in Chinese patients undergoing coronary angiography was significantly associated with low serum 1,5-AG and this effect was stronger in patients with diabetes. These results add to previous evidence highlighting the use of 1,5-AG as an effective glycometabolic marker. In another study using a transcriptomics approach to identify novel biomarker, Yu et al. (2022) used 10 human carotid atherosclerotic plaque samples from diabetic and non-diabetic patients to sequence coding RNA, non-coding RNA and identify potential biomarkers and RNA regulatory pathways associated with plaque formation and diabetes. Many of the genes and all RNA regulatory pathways associated with carotid atherosclerotic plaques in diabetic patients had not been reported, thus providing a platform for further studies validating these novel potential biomarkers.

Using established, already existing datasets to test hypothesis and build analytical models has proven to be a useful predictive tool in understanding disease development. Yang et al. (2022) used 2 high-quality critically ill databases to screen intensive care unit patients with both heart failure and diabetes with the goal of developing a composite indicator of predicting hospital mortality for patients. These patients have worse prognosis and higher mortality than patients with either heart failure or diabetes alone. A machine learning model was used to find indicators associated with hospital mortality for patients with both diabetes and heart failure, resulting in the creation of a novel composite indicator that combines three major attributes related to mortality risk [Acute Physiology Score (APS) III, Sepsis-related Organ Failure Assessment (SOFA), and Max lactate]. Studies such as these that use existing data to create novel disease outcome predictors with better clinical value are extremely valuable and have potential to help reduce mortality risk.

As our knowledge base increases, both technically and conceptually, there is a need to reevaluate existing practices and guidelines. Although CVDs are the result of a combination of risk factors, current guidelines do not account for the multitude of risk factors involved, nor do they take into account the heterogeneity among patients. As indicated by Li et al. (2022), the paradigm is shifting to ensure the intensity of treatments adequately match the risk level. A new risk stratification was applied to a Chinese cohort of 9,944 patients with atherosclerotic CVD (ASCVD). Among patients in various subgroups of ASCVD, worse outcomes were significantly associated with diabetes thus implicating a need to adjust ASCVD treatment in diabetic patients.

Finally, in a comprehensive review, Al Kury et al. (2022) emphasized the need for more studies focused on diabetes-induced sinoatrial node remodeling. Sinoatrial node dysfunction results in slow heart rate, eventually resulting in high blood pressure and coronary heart disease. Of note is the fact that chronic treatments for diabetes (including metformin, insulin, and rosiglitazone) adversely affect the sinoatrial node, and thus negatively impact cardiac function. Therefore, more adequate therapies are need for diabetic patients who also have impaired cardiac function.

Overall, the above-mentioned articles published in the second volume of this Research Topic highlight the broad scope of research being conducted to advance our technical and conceptual understanding of the interconnectedness of obesity, diabetes, and CVDs. We continue to expand our understanding of the cellular, molecular, and pathological processes involved in these chronic diseases, applying what we know to advance the discovery of novel biomarkers and therapies. However, it is clear there is much work to be done, data gaps to be filled and discoveries to be made.

## Author contributions

JY and LC made the first draft. MR, JY, YX, and LC revised and approved the final submitted version.

## Conflict of interest

The authors declare that the research was conducted in the absence of any commercial or financial relationships that could be construed as a potential conflict of interest.

## Publisher’s note

All claims expressed in this article are solely those of the authors and do not necessarily represent those of their affiliated organizations, or those of the publisher, the editors and the reviewers. Any product that may be evaluated in this article, or claim that may be made by its manufacturer, is not guaranteed or endorsed by the publisher.
